# Prediction of Immunomodulatory potential of an RNA sequence for designing non-toxic siRNAs and RNA-based vaccine adjuvants

**DOI:** 10.1038/srep20678

**Published:** 2016-02-10

**Authors:** Kumardeep Chaudhary, Gandharva Nagpal, Sandeep Kumar Dhanda, Gajendra P. S. Raghava

**Affiliations:** 1 Bioinformatics Centre, Institute of Microbial Technology, Chandigarh, India

## Abstract

Our innate immune system recognizes a foreign RNA sequence of a pathogen and activates the immune system to eliminate the pathogen from our body. This immunomodulatory potential of RNA can be used to design RNA-based immunotherapy and vaccine adjuvants. In case of siRNA-based therapy, the immunomodulatory effect of an RNA sequence is unwanted as it may cause immunotoxicity. Thus, we developed a method for designing a single-stranded RNA (ssRNA) sequence with desired immunomodulatory potentials, for designing RNA-based therapeutics, immunotherapy and vaccine adjuvants. The dataset used for training and testing our models consists of 602 experimentally verified immunomodulatory oligoribonucleotides (IMORNs) that are ssRNA sequences of length 17 to 27 nucleotides and 520 circulating miRNAs as non-immunomodulatory sequences. We developed prediction models using various features that include composition-based features, binary profile, selected features, and hybrid features. All models were evaluated using five-fold cross-validation and external validation techniques; achieving a maximum mean Matthews Correlation Coefficient (MCC) of 0.86 with 93% accuracy. We identified motifs using MERCI software and observed the abundance of adenine (A) in motifs. Based on the above study, we developed a web server, imRNA, comprising of various modules important for designing RNA-based therapeutics (http://crdd.osdd.net/raghava/imrna/).

Mammalian innate immunity has dedicated sensor and effector proteins to combat viral invasion. Interferons were the first class of molecules to be discovered, acting as armor for extracellular defense against viruses^1^. Much later, Janeway postulated the theory of pattern recognition, propounding the existence of receptors in the host cells that recognize molecular structures associated with pathogens named pathogen-associated molecular patterns (PAMPs). To be recognized as ‘foreign’, the PAMPs should be present in the pathogens but not in the host cells^2^. These PAMPs are recognized by dedicated receptors called pattern recognition receptors (PRRs)^3^. In mammalian systems, the PRRs have been broadly categorized into two classes. The first category is that of membrane-bound receptors that includes the Toll-like receptors (TLRs) and C-type lectin receptors (CLRs), while the second category is composed of the cytoplasmic sensors, for example, NOD-like receptors (NLRs), RIG-I-like receptors (RLRs) and a growing list of cytosolic nucleic acid sensors^4^ (23846113). A schematic depicting the localization of major mammalian PRRs within the cell has been shown in Fig. 1.

In case of a few viral infections, cellular defense system recognizes the pathogen from its RNA sequence. In other words, RNA sequences also serve as PAMP for viral infections. It was shown that a firm innate defense against bacterial RNA involved cytosolic detection of bacterial mRNA^5^. This study showed that the mammalian innate immune system can detect microbial viability by virtue of the viability-associated PAMPs (vita-PAMPs) present in the pathogen and recognized prokaryotic mRNA as a vita-PAMP. The authors thus suggested the inclusion of vita-PAMPs like mRNA in vaccine formulations to supplement the safety of dead vaccines with the superior attributes of live vaccine^5^.

Numerous receptors for RNA in the cytosol of human cells have been discovered. In addition to the diversity of types of RNA ligands of these receptors, the overlap of the Pathogen-associated molecular patterns in the ligands of these receptors is also enigmatic^6^. Single-stranded RNA from microbial sources is known to bind endosomal Toll-like receptors 7/8 (TLR-7/8)^7^,^8^ and the cytosolic receptors RIG-I 9 and NOD2^10^. Despite the knowledge that ssRNA binding with TLR-7/8 is sequence-dependent, sequence characteristics of such binding are not well defined^11^. Proclivity to the uridine or guanine-rich sequences including the motifs UG and UGU has been reported in case of TLR-7/8 ligands^7^,^8^. Another study demonstrates the concomitance of immunostimulatory property of RNA with AU-richness in the sequence^12^.

siRNAs are RNA oligonucleotides that spearhead the RNA Interference (RNAi) technology. Our cellular sensors may recognize these siRNAs as foreign RNA and may activate the immune system, which is undesirable and may cause immunotoxicity^13^,^14^. If such off-target immunotoxicities are uncontrolled, the clinical results of administering such RNAi molecules could be catastrophic^15^. On the contrary, in diseases like cancer and viral infections, immunostimulatory properties of siRNA could augment the therapeutic effect that relied solely on gene silencing ability. Such bifunctionality has been achieved using sequence-based modifications^16^,^17^. These studies suggest importance of single-strand RNA (ssRNA) motifs in mediating immune response due to RNA species. Previously, a method has been patented (EP1764107A1), which classifies the ssRNA based on its immunostimulatory activity.

In this study, we collected and compiled ssRNA oligoribonucleotide sequences (ORNs) whose immunomodulatory potential has been tested experimentally. Using these experimentally validated siRNAs, we developed sequence-based models for discriminating immunomodulatory oligoribonucleotides (IMORNs) and non-immunomodulatory oligoribonucleotides (non-IMORNs). The experimentally validated IMORNs have been picked up from literature, patents, and RNAimmuno^18^ database. Finally, A web server has been developed for providing comprehensive information about a given RNA sequence in terms of its immunomodulatory potential. This server will facilitate the scientific community in designing an ssRNA sequence with desired properties.

## Results

### Composition Analysis

**Figure 2:**
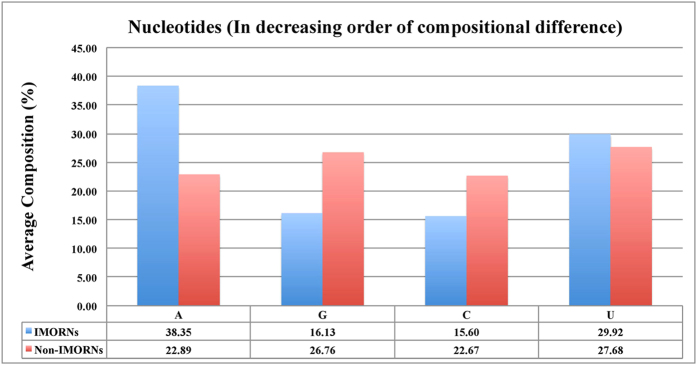
Comparison of Mononucleotide Composition between immunomodulatory and non-immunomodulatory RNAs.

**Figure 3:**
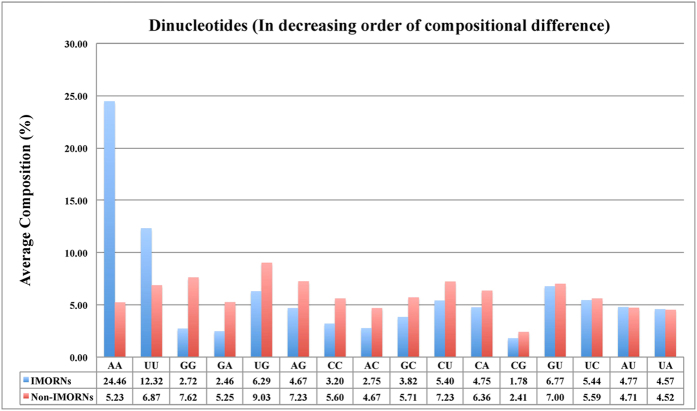
Comparison of Dinucleotide Composition between immunomodulatory and non-immunomodulatory RNA.

**Figure 4:**
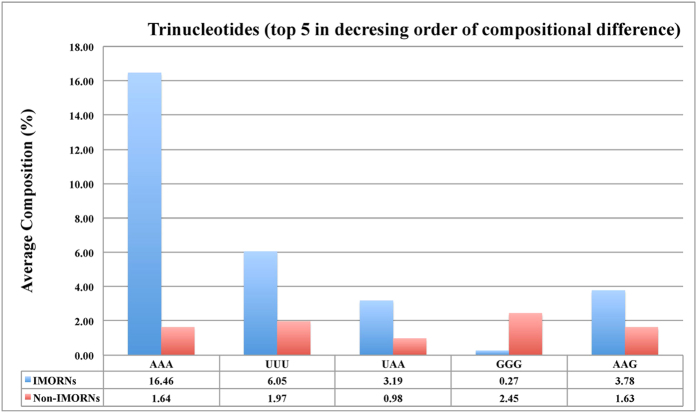
Comparison of Trinucleotide Composition between immunomodulatory and non-immunomodulatory RNA.

In order to understand the nucleotide bias in IMORNs and non-IMORNs, we computed and compared the nucleotide compositions of two types of sequences. Student’s t-test between mononucleotide composition of IMORNs and non-IMORNs revealed significant compositional differences in the cases of Guanine (G) and Adenine (A) (Fig. 2 and Table S1). This was further reflected in the dinucleotide composition analysis where 3 out of top 4 motifs (AA, UU, GG, GA), abundant in IMORNs, contain these two mononucleotides (Fig. 3 and Table S2) when sorted on the basis of absolute value of difference in the mean compositions in IMORNs and Non-IMORNs. Similarly, we identified trinucleotide motifs (e.g., AAA, UUU, UAA, GGG and AAG) that have maximum difference in mean values between the negative and positive sequences (Fig. 4 and Table S3); majority of them have G and/or A. We extended our analysis to higher-order composition and identified tetranucleotides and pentanucleotides with maximum difference in mean values among immunomodulators and non-immunomodulators (Tables S4 and S5). The top 5 tetranucleotides identified in this manner were AAAA, CCCC, AAAG, CAAA and UAAA while the top 5 pentanucleotides were AAAAA, CCCCC, AAAGC, CAAAA and GCAAA. In both of these cases, most of the topmost motifs were A-rich. These observations indicated that the composition of RNA sequences could be used to classify IMORNs and non-IMORNs.

### MERCI motifs

The role of motifs and patterns is crucial to discriminate and classify the biological sequences. Therefore, we have also tried to identify exclusive motifs in IMORNs and non-IMORNs. The Motif—EmeRging and with Classes—Identification (MERCI) program^19^ is a powerful software that can discover motifs by comparing two types of datasets. In this study, we used MERCI with different gap lengths (no gaps, 1, 2 or 5 gap maximum length) for discovering motifs in our positive and negative datasets. It was observed that most discriminative motifs were found when maximum gap length allowed was 5 (Table S6). The best motif was “AAA-AA-AA-A” in the positive dataset that was observed in 270 IMORNs (Table 1). On the other hand, the best motif in negative dataset was “C-G-G-G-GG-G” with the coverage of 81 non-IMORNs.

### Two Sample Logos

**Figure 5:**
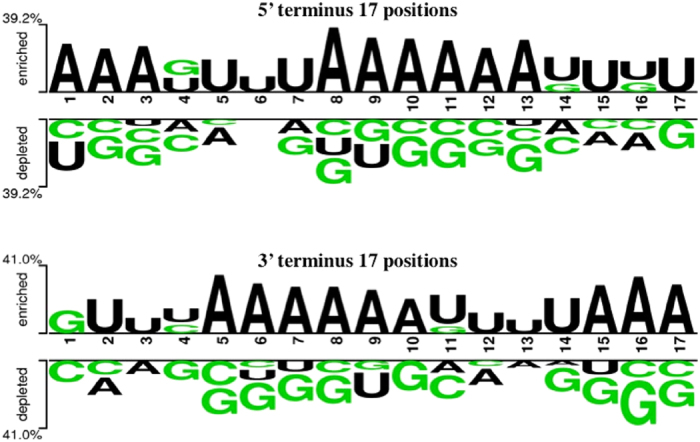
Two-Sample Logo of the 17 positions in the 5′ and 3′ termini of the immunomodulatory (enriched) and non-immunomodulatory (depleted) RNA sequences.

In order to visualize the position-specific preference of mononucleotides in IMORNs, we obtained the Two Sample Logos (TSL)^20^ depiction using the tool available at http://www.twosamplelogo.org/cgi-bin/tsl/tsl.cgi. Creation of TSL requires fixed length of all the sequences. Since the sequence length in the dataset varied from 17 to 27, we created a 17mer TSL separately for 5′ and 3′ termini of the sequences (Fig. 5). TSL depiction (Fig. 5) clearly shows the dominance of Adenine (A) in the IMORNs while Guanine (G) is more abundant in non-IMORNs. This corroborates well with the compositional as well as MERCI motif analysis, where motifs rich in A were found to be significantly different in occurrence between the positive and negative datasets.

### Prediction of Immunomodulatory RNA

#### Composition-based models

The compositional analysis revealed that the average composition of certain mononucleotides, dinucleotides, trinucleotides, tetranucleotides and pentanucleotides is significantly higher in IMORNs in comparison to non-IMORNs. This means composition can be used as a feature for discriminating these two classes of RNA sequences. Thus, we developed support vector machine (SVM)-based models using composition of RNA sequences as input feature, for predicting IMORN sequences. To evaluate the performance of these models, we used internal validation and external validation techniques. Additionally, we also used a sampling approach for calculating the average performance of models on internal and external datasets. In the case of internal validation, we computed the performance of models on training-testing datasets (IMrnaTT) using five-fold cross-validation technique. The average performance of our models on internal datasets is shown in Table 2. The performance of models improved with the order of composition (from mononucleotide to pentanucleotide) and achieved the maximum average Matthews Correlation Coefficient (MCC) of 0.86 with 93.00% average accuracy using the model based on pentanucleotide composition.

#### Models developed using binary vector

In case of binary representation, we represented a mononucleotide with a vector of length four, it means a sequence of length *N* will be represented by a vector of length *N* × *4*. In the present study, we have RNA sequences of varied length thus it is difficult to represent them using fixed length vector (a requirement of SVM). To overcome this limitation, we used fixed length of mononucleotides from the termini of RNA sequences for developing binary-based models. First, we used 17 nucleotide positions from 5′ terminus of RNA sequences for developing the model using binary profile of these mononucleotides; these mononucleotides were represented by a vector of length 68 (*17* × *4*). Similarly, we developed models using 3′ end of RNA sequences. Finally, we developed models using both the ends of the sequences; our final model used both 5′ and 3′ termini sequences, achieving a mean accuracy and MCC of 90.44% and 0.81, respectively.

#### Model based on hybrid features

We also checked a hybrid model having all features of trinucleotide composition along with the binary profile of 5′ and 3′ termini taken together for performance. The accuracy, in this case, was 91.79% while MCC was 0.84, both being mean values of those obtained in each of the 10 sampling cases.

#### Performance of models on independent dataset

The performances of the composition, binary as well as hybrid models were evaluated on the independent datasets (ImrnaID) also called external validation datasets. It is important to validate any model on independent datasets as these have not been used in training or testing of the models. As shown in Table 3, our models performed equally well on independent dataset and achieved the mean accuracies in the range of ~79% to ~93% using different types of nucleotide compositions. Similarly, our hybrid model achieved a mean accuracy of 92.54% on independent dataset. This means our models are not over optimized, as the performance of these models is comparable in both, the internal and the external validation.

### Feature Selection

WEKA^21^ was used for selecting relevant features that may significantly contribute to the prediction. We selected the best features from various types of nucleotide compositions and used these selected features for developing models (Table S7). In the case of trinucleotides, WEKA selected 20 best features out of 64 and the models based on best features achieved the mean MCC of 0.75 and 0.76 on training-testing and independent datasets respectively. In case of pentanucleotides, WEKA selected 29 features out of the 1024 features and achieved poor performances on both, IMrnaTT as well as IMrnaID datasets. We also selected best features from all composition-based features (e.g., mononucleotide, dinucleotide, trinucleotide, tetranucleotide, pentanucleotide) and got 81 features. These 81 features were used for developing prediction models and achieved the maximum MCC of 0.84 and 0.76 on training-testing (IMrnaTT) and independent datasets (IMrnaID) respectively. It is clear from the above analysis in this study that feature selection was not advantageous in this study.

### Structure Analysis

Previous studies have implied the role of RNA secondary structure in immunostimulatory activity^16^,^22^. We performed a comparative analysis of predicted RNA secondary structures of IMORNs and non-IMORNs in our dataset using the RNAfold program available in the ViennaRNA package^23^. First we predicted the secondary structure of both the IMORNs as well as the non-IMORNs and then we compared the structures between the two classes. The RNAfold program predicts extended open structure (no stem-loop) for 353 out of 602 IMORNs (58.64%) and 128 out of 520 non-IMORNs (24.61%). The observation that more than half of the IMORNs have linear structure while only about a quarter of the non-IMORNs have extended linear structure might indicate that linear structures are preferred in IMORNs. For observing the dependence of RNA secondary structure on the length of the RNA sequences in our datasets, we divided the positive and the negative datasets into the bins of lengths 17–20, 21–23 and 24–27 as shown in Table 4. We observed that most of the IMORN sequences have length in the range 17–20 while the non-IMORNs are mostly 21–23 nucleotides long. Moving from the bin of shorter length to the longer ones in IMORNs, the proportion of sequences with regular secondary structure is increasing as compared to the sequences with linear structure. In contrast, the proportion of regular structures is greater across all the length bins in the non-IMORNs. In the length range 24–27, only 2 out of 22 non-IMORNs (9.09%) have linear structures while 48 out of 109 IMORNs (44.04%) are in linear conformation. Thus, looking at the proportion of sequences having stem-loop structures in both IMORNs as well as non-IMORNs, it appears that the tendency to form stem-loop secondary structure is proportional to the length of the RNA sequence. Yet, the inclination to form secondary structure appears to be more in non-IMORNs compared to the IMORNs as evident from the length bin 24–27 (Table 4). Next, we evaluated the occurrence of uridines in the stem-loop structures in IMORNs and non-IMORNs. In both the cases and across all the length bins, more stem-loops have uridines than those lacking, though their proportion seems to be increasing with length in case of IMORNs while in non-IMORNs, uridine-containing stem-loops are decreasing as the length of the sequences is increasing.

### Web interface for imRNA

In order to provide service to the scientific community, we developed a web server that implements the above prediction models. This web server is available for free use at http://crdd.osdd.net/raghava/imrna/. It has a number of modules; here we provide a brief description of two major modules of this server.

#### Designing of vaccine adjuvants

This module allows the users to design RNA-based vaccine adjuvants with desired immunomodulatory potentials. It has three sub-modules developed for performing various predictions on ssRNA. First sub-module allows the users to perform virtual screening of RNA oligonucleotides where the server predicts immunomodulatory potential of each oligonucleotide in a given library. Secondly, the server allows the users to design analogs by generating all the possible analogs (single site substitutions) of an RNA oligonucleotide and predicting the class (IMORN or non-IMORN) of each analog. This way, the user can select the most appropriate analogs with desired immunomodulatory potentials. Thirdly, the server facilitates the users in the identification of immunomodulatory regions (RNA oligonucleotides) in a given RNA sequence. In order to provide useful information about predicted RNA oligonucleotides, the server also computes the structure of these RNAs using RNAfold program available from the ViennaRNA package 2.0^23^ visualized in the VARNA^24^ applet.

#### Immunotoxicity of siRNAs

For the users working in the field of siRNA-based therapeutics, we developed a module that facilitates the users in predicting toxicity of siRNA sequences. In cases where the immunomodulatory ability of siRNA proves to be a disadvantage, the user might be in search of siRNAs with minimal immunotoxicity. First sub-module allows the users to submit a library of siRNA sequence for predicting immunotoxicity or immunomodulatory capability of each siRNA. The server also computes siRNA efficacy of query siRNAs using desiRm^25^ software developed previously by our group. Thus, the user may select siRNAs with desired efficacy and toxicity from a given set of siRNAs. Another sub-module allows the users to design siRNA with desired efficacy and immunotoxicity. Under this sub-menu, the server generates a large number of analogs corresponding to the siRNA submitted by a user followed by the prediction of efficacy and immunotoxicity of each analog.

## Discussion

In the past, meticulous efforts have demonstrated the importance of understanding the immunostimulatory potential of RNA sequence for designing better therapeutics. It has been shown previously that combining gene silencing with virus-like immunostimulation of RNAs (two-pronged strategy) can be used for effective treatment of cancer and viral infections^26^. Gantier *et al.*^16^ have demonstrated that sequence-based rational approaches can confer immunomodulatory potential to a siRNA without affecting its gene silencing activity. On the other hand, Judge *et al.*^27^ identified the immunomodulatory motifs in siRNAs and showed that altering the nucleotide sequence could subside the immunomodulatory effect considered to be a siRNA-associated immunotoxicity. Thus, while the immunomodulatory potential of siRNA is a desirable therapeutic effect in some cases, it is an associated immunotoxicity in others. imRNA provides an *in silico* platform that has modules for both the purposes – enhancing immunological therapeutic ability of ORNs as well as diminishing the siRNA immunotoxicity.

In diseases like cancer and viral infections, immunostimulatory properties of siRNA could augment the therapeutic effect that relied solely on gene silencing ability. Such bifunctionality has been achieved using sequence-based modifications^16^. The study showed an increase in immunostimulatory effect on introducing a non-pairing U-rich (uridine rich) bulge within the passenger strand of siRNA enhanced the immunostimulatory effect remarkably. In another study, a thermodynamic investigation into the hybridization strength between two strands of siRNA revealed a negative correlation between duplex strength and immunostimulation^17^. The authors suggest that increasing the duplex strength by introducing CG or GC in the sequence retards the release of immunostimulatory single strand and have proposed a model in which the U-rich strand of the siRNA duplex and the ease of its release as a single strand is the major source of causing immunostimulation mediated by TLR-7/8. The detailed investigation into the mechanism of siRNA immunostimulation proposed a model in which the siRNA duplex undergoes endosomal denaturation leading to two single strands of RNA and the U-rich sequences among these single-stranded species activate TLR 7/8 17. A comparative study also showed that siRNA sequences having immunostimulatory motifs are more effectively recognized by the innate immune system as single-stranded molecules rather than their duplex counterparts^28^. Such studies provide a strong indication towards the importance of ssRNA motifs in mediating immune response due to RNA species. This forms the theoretical basis for the need of methods for predicting whether a given ssRNA oligonucleotide would be immunostimulatory or not. Such a method could also help in selecting or rejecting siRNA sequence alternatives that are immunomodulatory or non-immunomodulatory depending on the case whether this attribute is considered to be a therapeutic potential or a toxic effect.

In our study, we found that the positive (immunomodulatory) sequences are enriched in A-rich motifs as compared to the negative sequences where the G-rich motifs prevail. The primary reason for this result is that our negative dataset is made from the circulating miRNAs found in the body fluids. On comparison of the nucleotide composition between the positive and the negative dataset, the difference in the U composition between positive and negative sequences was found to be least significant (greatest p-value, Table S1) among the four types of nucleotides. Previous literature firmly propounds the importance of U-richness in RNA immunostimulation. In contrast, Jurk *et al.*^29^ have reported non-uridine rich sequences that have a strategically located single uridine in a particular sequence-context mediating immunostimulation through TLR 7. Even Gantier *et al.* 2008^22^ also found that the localization of uridines in the stem-loop of ssRNA structure is important in addition to the previous claims of U-richness for RNA immunostimulation. Thus, rather than being contrary to previous studies reporting U-richness, our result of A-rich motifs found in the IMORNs might be referring to the sequence context accompanying the uridines in the sequences. More than 98% (593 out of 602) of the positive sequences have atleast one uridine. Additionally, it has also been proposed that TLR 8 preferentially senses loose regions of single strand secondary structures containing uridines^16^ that might be provided by A-rich motifs as sequence context.

Gantier *et al.* 2008^22^ in their experimental procedure showed that introduction of uridines in the stem-loop structure (predicted by mFOLD program) of an ssRNA transformed it from a non-stimulatory sequence to a TNF-α inducing sequence thus suggesting a possible role of RNA secondary structure in immunostimulation. In our analysis, we found that more than half of the IMORN sequences were predicted to have open linear structures while this was the case in only a quarter of the non-IMORNs. Gantier *et al.* 2010^16^ have proposed that TLR 8 preferentially recognizes loose secondary structure but this recognition is affected by the neighboring intramolecular secondary structure. When we distributed the IMORNs into lengthwise bins and observed the secondary structure occurrence, we found that starting from more number of linear structures in shorter sequences, increase in length has a tendency to favor stable secondary structure (stem-loop) formation partly in agreement with the experimental study. Similarly, the proportion of uridine containing stem-loop structures out of the total stem-loops in IMORNs (68.37%) does not seem to be very different from their proportion in non-IMORNs (72.97%). Yet, their occurrence in lengthwise distribution bins shows an opposite trend. With increase in length, IMORNs seem to favor uridine-containing stem-loops while the non-IMORNs show decreasing proportions of such stem-loops. Thus, the RNA secondary structure of IMORNs seems to be length-dependent.

One of the challenges in this study was to compile data from the literature for developing prediction models. The quality of any model depends on datasets used for developing models. Thus, we systematically collected 602 immunomodulatory ssRNA sequences from patents and RNAimmuno database^18^. Similarly, we collected 520 miRNAs that have been found to be in circulation in the human body from the database miRandola^30^. We assigned these miRNAs as non-immunomodulatory RNA sequences based on the assumption that being in circulation in the human body fluids, these have little or no immunomodulatory effect. One may argue why these miRNAs have been assigned non-immunomodulatory without experimental validation. We agree with argument, but we do not have better assumption for creating negative dataset, which is must for developing classification model. Another limitation is the moderate size of the positive dataset; a bigger dataset is needed to develop more reliable models. With larger datasets, we believe in future better and more reliable models will be developed. This study may be considered an initiative towards the development of models for predicting immunomodulatory ssRNA sequences.

## Conclusion

There are numerous resources and *in silico* tools for predicting immunogenicity and antigenicity potential of peptides^31^,^32^,^33^,^34^,^35^,^36^,^37^,^38^. In contrast, limited efforts have been made to understand the immunomodulatoy potential of RNAs. This study is an attempt in this direction where *in silico* models have been developed to predict the immunomodulatory effect of RNA sequences. These models can be used for designing RNA sequences with desired immunostimulatory effects. It means that the user may increase or decrease immunomodulatory property of an RNA sequence by mutating certain nucleotide positions. The choice remains with the scientific community as to how and for what purpose they use our web server, either for designing better adjuvants or for reducing the toxicity of their RNA sequences. A major application of these methods is the design of RNA sequences that manifests gene silencing and elicits immune responses concomitantly. To implement this application, a separate module has been provided in the web server that suggests whether the query siRNA sequences would be immunomodulatory or not, apart from predicting its gene-silencing efficacy.

## Methods

### Datasets

In the present study, we took ssRNA sequences that were experimentally shown to be immunomodulatory. The positive sequences taken have been adjudged to be the immunomodulatory ssRNAs in different studies through various assays like release of cytokines IFN-α and TNF-α from human PBMCs (Peripheral Blood Mononucleur Cells)^8^,^22^ upon administration as a single-strand complexed with DOTAP. Sioud, 2006 has found that adherent PBMC respond more to single-stranded siRNAs (ss siRNAs) in comparison to the siRNA duplexes and addition of ss siRNAs to siRNA duplexes enhanced the immune response^28^. Administration with DOTAP ensured delievery of ss siRNAs into the endosome which is a compartment harboring the sensors of ssRNAs namely TLR 7 and 8.

We found 617 such sequences of ssRNA in 4 patents available in the literature of which 493 sequences had length from17 to 27 nucleotides. RNAimmuno^18^ is a collection of immune side effects of RNA molecules. From this database, 232 ssRNA sequences were retrieved that were reported for immunological side effects of which 166 sequences were in the length range of 17–27. From these two sources taken together, we obtained 602 unique sequences having nucleotide length range of 17–27, which were used for building the positive dataset. Only those studies from the RNAimmuno database and only those patents where the RNA species examined was single-stranded including the ss siRNAs were taken for constituting the positive dataset. Further, only the nucleotide sequences were considered and this study did not include the chemical modifications at the termini or the backbone of the sequences to build the prediction models. In the absence of RNA sequences experimentally shown to be non-immunomodulatory, we collected the miRNAs from the miRandola^30^ database. miRandola is a database of extracellular circulating miRNAs in human biological fluids like serum, plasma, saliva and urine. Since the source of these miRNAs is the extracellular fluids of healthy and cancerous human tissues, these RNA sequences were assumed to be non-immunomodulatory and were used to constitute the negative dataset. A total of 520 miRNAs in miRandola were mapped onto the miRBase^39^,^40^ database to retrieve the sequences. Our main dataset IMrnaDS contains 602 positive (or immunomodulatory) sequences called IMORNs, and 520 negative (non-immunomodulatory) sequences called non-IMORNs.

**Figure 6:**
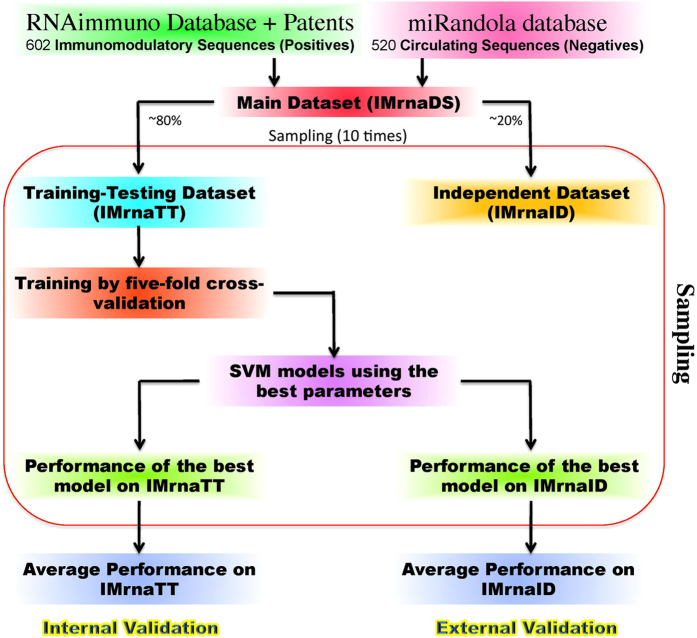
Flow diagram showing the sampling method used for evaluating the prediction models.

In this study, we performed both internal and external cross-validation, as well as sampling approach for computing unbiased performance of our models. First we created a training-testing dataset called IMrnaTT that constitutes ~80% sequences (482 positive and 416 negative sequences) of main dataset IMrnaDS. We used the remaining sequences for creating independent dataset IMrnaID containing ~20% (120 positive and 104 negative sequences) of the main dataset IMrnaDS (Fig. 6).

#### Internal Cross-Validation

The training-testing dataset, IMrnaTT, was subjected to five-fold cross-validation in which the dataset is divided into five parts. In each of the five rounds of evaluation, the model is trained on four parts and tested on the left out fifth part. In this manner, each part has been tested once. Finally, we computed overall performance of a model on whole IMrnaTT dataset. To optimize our SVM models, we tried various combinations of SVM parameters (like kernel, cut-off values) for getting maximum performance. We tried most of the SVM parameters or combination of SVM parameters for developing and evaluating the performance of each model on IMrnaTT dataset. Finally, we identified the SVM parameters that gave best performance of an SVM model on our training-testing dataset IMrnaTT. The best performance of the model was found from the above process called internal validation.

#### External Validation

In case of internal validation, we optimized the performance of our models by trying all possible combinations of SVM parameters and identified the best SVM parameters. We developed an SVM model on training-testing dataset IMrnaTT using these best SVM parameters. We evaluated the performance of this SVM model on the independent dataset IMrnaID, this process being called external validation of a model.

#### Sampling

The process of creating the training-testing dataset (IMrnaTT) and the independent dataset (IMrnaID) from the main dataset (IMrnaDS) was repeated ten times. Each time, the sequences for IMrnaTT were randomly selected from IMrnaDS, and the remaining sequences were included in IMrnaID (a procedure similar to *Monte-Carlo Sampling*). Finally, we evaluated the performance of our models using various features on training-testing and independent dataset as described in the above sections. This process gave 10 performance values using internal and 10 performance values using external validation from 10 rounds of sampling. We computed the mean and standard deviations of these performance values on IMrnaTT and IMrnaID datasets provided in the Tables 2 and 3.

### Compositional analysis

Compositional features of nucleotide sequences have been used for analysis and prediction of different classes of non-coding RNAs^41^. In our study too, we used the compositional features of oligonucleotides such as mono-, di-, tri-, tetra- and pentanucleotide compositions. The vector size for mono-, di-, tri-, tetra- and pentanucleotide compositions comes out to be 4, 16, 64, 256 and 1024, given that the mononucleotide types are 4 in number.

### Motif-based analysis

Motif identification can provide functionally important aspects of biological sequences^19^. MERCI program was used to find motifs that could effectively distinguish positive sequences from the negative ones or vice versa. The program can identify motifs exclusive to any of the positive and negative dataset or those more prevalent in one of these and allowed to a defined lower extent in the other. In our case, the MERCI program compared immunomodulatory RNA (positive) sequences with non-immunomodulatory RNA (negative) sequences for finding the motifs.

### Implementation of support vector machine

In this study, we developed SVM-based classifiers, which are efficient classifiers heavily used in previous studies including the work on RNA classification^41^. Compositional features calculated in these categories, *viz.* mononucleotide, dinucleotide, trinucleotide, tetranucleotide and pentanucleotide composition were calculated for each of the sequences in the dataset. These features were used for developing SVM-based prediction models separately for each of the categories using the software SVM^*light*^ 42. Apart from this, models based on the binary composition were also used for prediction. Binary notations of A (1,0,0,0), C (0,1,0,0), G (0,0,1,0) and U (0,0,0,1) were used to generate the concatenated profile derived from 17 nucleotide positions taken from the 5′ end and 17 nucleotides from the 3′ end; and were used for SVM predictions. Additionally, hybrid models using trinucleotide as well as binary composition or by using the combination of trinucleotide composition and binary composition were also developed to investigate the contribution of nucleotide positions in a sequence to the immunomodulatory property.

### Feature Selection

WEKA^21^ is a package that helps to identify the features that contribute most significantly to the prediction models using various classifiers. In the categories, trinucleotide and pentanucleotide compositions, WEKA was used for identifying the important features (trinucleotide and pentanucleotide motifs in this study).

## Additional Information

**How to cite this article**: Chaudhary, K. *et al.* Prediction of Immunomodulatory potential of an RNA sequence for designing non-toxic siRNAs and RNA-based vaccine adjuvants. *Sci. Rep.* **6**, 20678; doi: 10.1038/srep20678 (2016).

## Supplementary Material

Supplementary Information

## Figures and Tables

**Figure 1:**
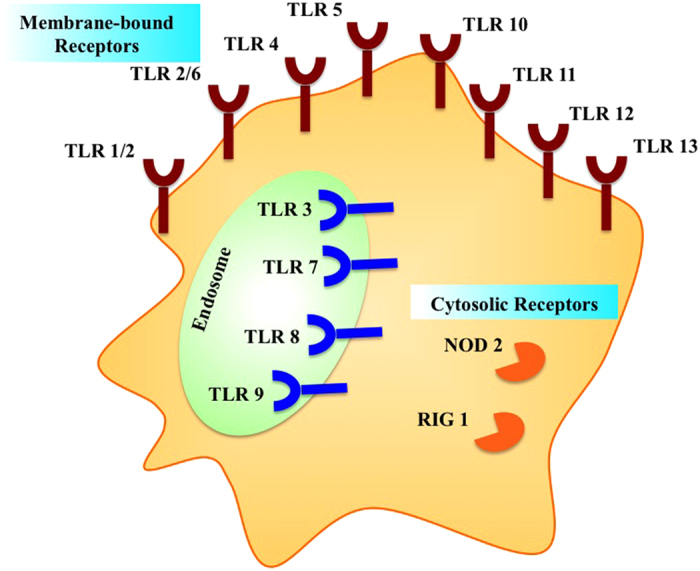
A diagrammatic representation of major mammalian pattern recognition receptors (PRRs) with their localization within the cell.

**Table 1:** Distribution of the positive and negative motifs with their coverage and maximum number of gaps allowed.

Positive motifs	MaximumGap length	Coverage	Negative motifs	MaximumGap length	Coverage
AAA-AA-AA-A	5	270	C-G-G-G-GG-G	5	81
AA-AAA-AA-AA	5	268	G-C-G-GGG	5	66
A-AAAA-AA-A	5	268	C-G-G-GGG	5	66
AAAAAA	0	267	C-C-C-GGG	5	62
AAA-G-A-AAA-A-G	5	266	G-C-GGG	2	61
AA-AAA-A-G-AA	2	194	C-G-GG-G	1	59
A-A-G-C-AAA	1	141	A-GC-G-GG-G	5	59
AA-G-C-A-AA	1	139	C-G-GGG	2	55
A-AA-G-G-A-A-A	1	103	G-C-G-GG	1	54
G-AAAA-A-A	1	98	AG-GC-G-AG	5	53

The motifs were extracted using MERCI program.

**Table 2:** Average performance obtained from internal five-fold cross-validation of the SVM models developed for predicting immunomodulatory potential of RNA sequence using different types of features.

Features (Composition)		Thres	Average Performance of Models			
Type	Vector size		Sen	Spec	Acc	MCC
MNC	4	0	78.01 ± 1.87	77.98 ± 1.09	78.29 ± 1.45	0.57 ± 0.03
DNC	16	0	88.84 ± 1.06	89.73 ± 1.11	89.25 ± 0.84	0.78 ± 0.02
TNC	64	0	91.83 ± 0.81	91.71 ± 1.09	91.83 ± 0.83	0.84 ± 0.02
TetNC	256	0	91.85 ± 0.51	90.70 ± 1.31	91.32 ± 0.71	0.83 ± 0.02
PNC	1024	0	93.96 ± 0.67	91.88 ± 1.41	93.00 ± 0.83	0.86 ± 0.02
5′ bin	68	0	89.00 ± 1.06	88.87 ± 0.79	88.94 ± 0.69	0.78 ± 0.01
3′ bin	68	0	89.48 ± 1.09	88.39 ± 1.13	88.82 ± 1.24	0.78 ± 0.02
5′ - 3′ bin	136	0	91.70 ± 0.60	88.99 ± 1.52	90.44 ± 0.89	0.81 ± 0.02
hybrid	200	0	91.20 ± 0.84	92.48 ± 0.85	91.79 ± 0.70	0.84 ± 0.01

**Thres:** Threshold, **Sen:** Sensitivity, **Spec:** Specificity, **Acc:** Accuracy, **MCC:** Matthews Correlation Coefficient, **MNC:** Monoucleotide Composition, **DNC:** Dinucleotide Composition, **TNC:** Trinucleotide Composition, **TetNC:** Tetranucleotide Composition, **PNC:** Pentanucleotide Composition, **5′bin:** Binary profile 5′, **3′bin:** Binary profile 3′, **5′-3′bin:** Binary Profile 5′-3′, **hybrid:** Binary Profile 5′-3′+Trinucleotide Composition.

**Table 3:** Average performance obtained from the external validation of SVM models on independent datasets developed using different types of features.

Features (Composition)		Thres	Average Performance of Models			
Types	Vector size		Sen	Spec	Acc	MCC
MNC	4	0	78.42 ± 5.26	79.71 ± 3.38	79.02 ± 2.44	0.58 ± 0.04
DNC	16	0	90.50 ± 3.60	89.42 ± 2.03	90.00 ± 2.43	0.80 ± 0.05
TNC	64	0	92.58 ± 2.27	88.65 ± 2.02	90.80 ± 0.82	0.82 ± 0.02
TetNC	256	0	92.83 ± 2.52	90.58 ± 2.47	91.79 ± 1.56	0.84 ± 0.03
PNC	1024	0	95.00 ± 2.39	91.73 ± 2.09	93.48 ± 1.49	0.87 ± 0.03
5′ bin	68	0	89.83 ± 2.88	88.85 ± 2.91	89.38 ± 1.83	0.79 ± 0.04
3′ bin	68	0	91.25 ± 3.00	85.96 ± 3.15	88.79 ± 1.57	0.78 ± 0.03
5′-3′ bin	136	0	93.92 ± 3.47	90.67 ± 3.82	92.41 ± 3.09	0.84 ± 0.06
hybrid	200	0	93.50 ± 3.23	91.44 ± 3.19	92.54 ± 2.34	0.85 ± 0.05

**Table 4:** Lengthwise structure analysis of IMORNs and non-IMORNs as predicted by the RNAfold program.

Length Bin	IMORNs				non-IMORNs			
	17–20	21–23	24–27	Total	17–20	21–23	24–27	Total
# of Sequences	425	68	109	602	44	454	22	520
# of Extended Linear Structure Sequences	273 (64.23%)	32 (47.05%)	48 (44.04%)	353 (58.64%)	11 (25.00%)	115 (25.33%)	2 (9.09%)	128 (24.62%)
# of Stem Loop Structure Sequences	152 (35.76%)	36 (52.95%)	61 (55.96%)	249 (41.36%)	33 (75.00%)	339 (74.67%)	20 (90.91%)	392 (75.38%)
Total # of loops in sequences	152	36	65	253	33	340	23	396
# of loops with at least one Uridine	81 (53.29%)	31 (86.11%)	61 (93.84%)	173 (68.37%)	26 (78.79%)	247 (72.65%)	16 (69.56%)	289 (72.97%)
